# Beyond biopolitics: the importance of the later work of Foucault to understand care practices of healthcare workers caring for undocumented migrants

**DOI:** 10.1186/s12910-021-00726-z

**Published:** 2021-11-27

**Authors:** Dirk Lafaut

**Affiliations:** Department of History, Archaeology/Art Studies, Philosophy and Ethics (HARP), Free University Brussels (VUB), Pleinlaan 2, 1050 Brussels, Belgium

**Keywords:** Healthcare worker, Migration, Clinical ethics, Health policy, Humanitarianism, Foucault, Biopolitics, Care of the self

## Abstract

**Background:**

Undocumented migrants experience multiple institutional and legal barriers when trying to access healthcare services. Due to such limitations, healthcare workers often experience ethical dilemmas when caring for undocumented migrants. This article aims to understand how individual healthcare workers who regularly take care of undocumented migrants deal with these dilemmas in practice. So far, the role of healthcare workers in this context has mainly been theorized through the lens of biopolitics, conceiving of healthcare workers as merely obedient instruments of humanitarian government or gatekeeping.

**Methods:**

Based on semi-structured, in-depth interviews and ethnographic observations with healthcare workers in Belgium, we explore how they ascribe meaning, reflect upon and give shape to care practices in relation to undocumented migrants. We use Foucault’s later work on care of the self to interpret the accounts given by the healthcare workers.

**Results:**

Healthcare workers in clinical roles exercise a certain degree of freedom in relation to the existing limitations to healthcare access of undocumented migrants. They developed techniques such as purposefully being inattentive to the undocumented status of the migrants. They also try to master their affective responses and transform their bodily attitude towards undocumented patients. They perform practical mental exercises to remind themselves of their role or position in the wider healthcare system and about their commitment to treat all patients equally. These techniques and exercises are inspired by colleagues who function as role models, inspiring them to relate in an ethical way to limitations in healthcare access. The developed care practices sometimes reproduce, sometimes transform the legal and institutional limitations to care for undocumented migrants.

**Conclusions:**

The findings nuance the biopolitical analysis regarding the role of healthcare workers in healthcare delivery to undocumented migrants that has been dominant so far. Theoretically this article provides a reconceptualization of healthcare ethics as care of the self, an ethical practice that is somewhat independent of the traditional professional ethics.

*Trial Registration* Medical ethics committee UZ Jette, Brussels, Belgium – Registration date: 18/05/2016 – Registration number: B.U.N. 143201628279.

**Supplementary Information:**

The online version contains supplementary material available at 10.1186/s12910-021-00726-z.

## Background

As in many European and North-American countries, undocumented migrants in Belgium have restricted access to healthcare [1]. Previous international research has reported a range of individual, institutional and legal barriers to public healthcare systems for undocumented migrants [2, 3]. They also experience arbitrariness in healthcare professionals’ attitude [4]. These barriers are partly overcome by networks of committed healthcare professionals, who work both in the public healthcare system and voluntary (humanitarian) organizations [4–6]. These healthcare professionals have developed pragmatic approaches to offer healthcare to undocumented migrants. They rely on informal networks, in order to share the responsibility for finding appropriate care and medical treatments [4, 5].

Healthcare workers are confronted with several challenges in their encounters with undocumented migrants. The available care services are generally limited and overwhelmed [3]. In Belgium, for example, research shows a clear concentration of patients with undocumented status in those clinics that have a more welcoming attitude [7]. Healthcare professionals in these services also face many ethical concerns and professional dilemmas due to restricted healthcare access. In the public sector, healthcare professionals are forced to decide if, and to what extent, they grant access to resources that are officially reserved for citizens. This dilemma has mostly been theorized as a conflict between human rights and deontological norms on the one hand, and legal and institutional requirements on the other [5, 6, 8]. In the medical humanitarian sector, efforts focused on alleviating suffering can be instrumentalized to control and govern migration flows [9, 10]. Moreover, humanitarian practices are assumed to have disempowering effects, as they represent undocumented migrants as vulnerable, passive and dependent [9, 11]. These ethical concerns related to humanitarianism have mainly been theorized through the lens of Michel Foucault’s biopolitics [9, 11].

Little research has been conducted on what individual healthcare professionals themselves consider to be ethical behavior when they face ethical dilemmas and concerns in their everyday work with undocumented migrants. This article provides an empirical study, exploring how restricted healthcare access for undocumented migrants is addressed by healthcare workers, both in the voluntary sector and in the public sector. We show that the ethical reasoning of healthcare workers is quite different from the moral theories that have been used to understand these dilemmas until now. This article moves away from these normative modes of theorizing and evaluates the ethical reasoning of healthcare professionals in terms of Foucault’s concept of “care of the self” [12, 13]. Theoretically, this contribution provides a reconceptualization of healthcare ethics, as an ethics that is somewhat independent of the traditional normative professional ethics.

## Theoretical framework

The French philosopher and social historian Michel Foucault described professional medicine as a field of knowledge applying norms to bodies. Knowledge of the body makes the body a target for correction, manipulation, and processes of normalization [14]. These processes can be focused on correcting individual bodies in accordance to norms and ideas of normality [14] But Foucault’s work became particularly influential when he developed the concept of biopolitics to describe how medical knowledge and processes of normalizations also were, or could be, applied to populations. This allowed the control and governing of populations by identifying certain groups of people as outside the normal range [15–17]. As a concept, biopolitics describes how the biological health and well-being of populations is intertwined with operations of political power.

The concept of biopolitics has been productively used in the human sciences. Amongst others, it has been used to understand the role of humanitarian care practices in the management of undocumented migrant populations in Europe [9, 11, 18]. Fassin [18] used the term “biopolitics of otherness” to indicate how the body of migrants has become of central importance for politics of immigration. Illness and allegedly universally recognizable bodily suffering offer legitimacy to undocumented migrants to claim residence status in case of medical regularization. Simultaneously, political asylum has been progressively restricted. In this way, physio-pathological abnormalities become the ground for citizenship of undocumented migrants. In this way, they come to perceive themselves as victims soliciting compassion [9, 18]. Biopolitics is also used to theorize how humanitarian care practices in border zones go together with the securitization of borders, and the governance of migration and mobilities [19]. Furthermore, the concept is used to describe how the right to access healthcare is the only right that is attributed to migrants, thus obscuring that all other rights are withdrawn [20]. In biopolitical analyses of humanitarianism, healthcare workers’ desire to do good becomes caught in strategic questions. Their intention to deliver care to the disadvantaged according to certain ethical norms or values is entangled with operations of power reinforcing the existing power imbalances.

This application of Foucault’s work, which relates to his earlier work, shows how subjects (such as healthcare workers) are manipulated by discourses, authorities or other powers for purposes of surveillance. In this classical model of power, power is something some people have and others not. In other words, “power imposes itself on us, and, weakened by its force, we come to internalize and accept its terms” [21]. However, in his later work (i.e. *The History of Sexuality*, vol. 2–3, late interviews, and posthumous published works) Foucault’s understanding of power became more complex.^1^ In his work on “care of the self”^2^ he noted that power is not just repressive, but also productive of social positions. The self becomes a subject through power relations, and as a subject seeks freedom from control by others, in a way that rearticulates these power relations [21–23]. In this work, Foucault defined ethics in terms of “care of the self”, an ethics which he described as:

“those intentional and voluntary actions by which men not only set themselves rules of conduct, but also seek to transform themselves, to change themselves in their singular being, and to make their life into an oeuvre that carries certain aesthetic values and meets certain stylistic criteria.” [13].

In this model of power, the self is reflexive of the power relationships that constitute the self, and is able to reshape these relationships [13]. This requires gaining self-awareness of one’s situation, and of how one has been affected and keeps being affected by power relationships. Such self-knowledge is reached through self-examination, reflexivity and self-knowledge. It allows to make “transformations that one seeks to accomplish with oneself as an object” [12] through “techniques of the self”. These practices involve some kind of permanent exercise in self-mastery and struggles to transform oneself (and the way powers affect the self) within a specific historical context [12, 26, 27]. Through these practices, subjects look for “… the stylization of an activity in the exercise of its power, and the practice of its liberty.” [13]. These practices can, but do not necessarily have to, be understood as attempts of subjects inventively to modify, resist or escape the way in which they are governed. This is expressed in Foucault’s notion of “counter-conduct” [28]. Counter-conduct does not necessarily mean a rejection of being governed in general, it involves less visible practices of resistance addressing the question of “how not to be governed *like that*” [29]. Counter-conduct does not address the structural injustices, but rather looks for less visible practices of resistance in settings that often appear ‘apolitical’ [29, 30].

The significance of Foucault’s work on “care of the self” has received some attention in bio-ethics literature [17, 31]. It is proposed that bioethics drawing on this work challenges traditional approaches to bioethics which focus predominantly on reason and autonomy [32]; it is argued that a model based on care of the self could enable healthcare workers to give greater weight to practice and the aesthetic [17, 31] However, only very few studies describe specific practices when it comes to understanding how healthcare workers engage in practices of the self. Randall et al. [33] studied how mental healthcare workers try to form themselves as an ethical subject when treating victims of sexual abuse. They show how these care workers engage with practices of self-mastery to disassociate themselves from prevailing medical and psychiatric conceptions of normality and abnormality. Munro [34] described, on an organizational level, the role of practical exercises in social movement organizations in transforming the subjectivities of their members. Guta et al. [35] described how HIV-researchers challenge themselves to work ethically beyond the requirements of the institutional ethics review boards. On the other hand, Shaw et al. [36] have been critical of the efforts of healthcare workers to engage in practices of ethical self-fashioning. They argued that such efforts of healthcare workers to provide culturally appropriate healthcare were insufficient in eliminating prejudice in healthcare services.

Scholars in the field of humanitarianism have also engaged with the concept of “care of the self”, but interpreted it in a way that is quite different from Foucault’s initial conceptualization. Malkki [37] described humanitarian work as care of the self, a practice to address the neediness of the caretaker, the giver… Thereby she described humanitarian work as a self-interested and self-humanizing practice, to claim personhood and to imagine oneself as part of something greater. Similarly, Givoni [38] argued that the concern for others in the humanitarian endeavor is narcissistic. Control over and surveillance of people in the global peripheries is achieved not only through care for endangered populations but also through care of the self, a process of ethical self-cultivation by means of which healthcare workers fashion themselves as more enlightened personae.

These existing studies within the field of bioethics and humanitarianism show that the conceptual development of the late work of Foucault is still very much in embryonic form. In contrast to the existing scholarship, this inquiry highlights the importance of practical exercises for self-mastery and self-transformation and explores its potential for conceiving of an alternative ethics for healthcare workers. We study the care practices of healthcare workers towards undocumented migrants. To this end, we use Foucault’s work on “care of the self” as a lens to describe how healthcare workers in this context form themselves as an active ethical subject, moving beyond humanitarian government and biopolitical processes of categorization.

## Methodology

This article is part of a wider research project concerned with how healthcare workers and undocumented migrants manage dilemmas related to accessing healthcare services in Belgium. Belgium has a federal legal framework, notably the law on ‘Urgent medical Aid’ (UMA), covering who should account for the provision of medical services to undocumented migrants. They can obtain a medical card, i.e. a three month permit to access regular public healthcare services, after undertaking a parallel administrative procedure via a physician and the Public Social Welfare Office (PSWO) in the municipality where they live. Once they have received a medical card, medical costs are covered.

The Belgian Advisory Committee on Bioethics [39, 40] repeatedly stated that “the administrative status of a stranger, regardless of the reasons for residence on Belgian territory, cannot have a negative impact on the delivery of medical care.” Nevertheless, research has shown these professional codes, as well as the above-mentioned legislation to be poorly implemented, evinced, amongst others, by the utilization rate, and the per capita expenditure, being far lower than Belgian residents [1]. The difficult healthcare access of undocumented migrants in Belgium cannot be disconnected from the progressive exclusion of undocumented migrants from welfare benefits (unemployment benefits, pensions, social housing, public health insurance or other forms of financial and material support) over the past 3 decades in Belgium. Although the law UMA formally provides an exception to this wider evolution, research shows a progressively more restrictive application of that law [41]. Research has shown that the application procedure for a medical card at the PSWO is also closely linked to governmental strategies of mobility control [41]. Moreover, circulating discourses of abuse, public discrediting of medical-humanitarian aid to asylum seekers and hunger striking undocumented migrants, discriminatory practices of individual healthcare workers as well as micro- and macro-economic exclusion mechanisms (i.e. the individual lack of financial resources and budgetary deficits of public hospitals due to austerity measures) all contribute to the impaired healthcare access of undocumented migrants in the public healthcare system [1, 3, 41]. In response to this structural inaccessibility a parallel network of medical humanitarian NGOs has emerged.

The data presented in this article are based on focused, multi-site ethnographic observations and on 45 semi-structured, in-depth interviews with healthcare workers in urban areas in Belgium. The observations informing this article were conducted by the author between September 2016 and September 2018 in two different sites where access to healthcare services was negotiated, notably in the emergency department in a Brussels hospital and in a reception centre that provides legal advice, day-shelter, and assistance to undocumented migrants. The level of participation during the observations varied according to the setting. Participants for the interviews were recruited by means of a purposive sampling technique in Brussels in order to represent different clinical professions (nurse, GP and specialist) and institutional settings (NGO-clinic, GP-practice, community health centre and hospital) (See 'Interview guide healthcare workers' in Additional file 1). All participants were health professionals who were frequently consulted by undocumented migrants. They are not necessarily representative of those working in the healthcare services as a whole. Written and verbal informed consent was obtained from all participants. Accordingly, all names are pseudonyms for reasons of confidentiality and anonymity. The research design was approved by the relevant medical ethics committee.

During the interviews and observations, we explored how healthcare workers adapted or changed their care practices as a consequence of state-imposed categorizations, distinguishing between documented and undocumented patients. We focused on which pragmatic approaches they developed and how these were limited by institutional policies, administrative requirements and professional guidelines. We asked them to explain how they evaluate their own approach and what meaning they ascribe to these care practices.

These data were analyzed using the qualitative software NVIVO 11. Coding was carried out in the language of the interview by the author. The quotes have been translated from the original language into English for this publication. To analyze the data, we distinguished the practices of responsibility of healthcare workers in different concrete relationships when they encounter obstacles to access healthcare for undocumented migrants. We analyzed how health care workers accept, assign and deflect responsibility for specific demands of care, and how this responsibility is negotiated with undocumented patients, colleagues and healthcare workers in other institutions. For the analysis informing this article, we specifically focused on how healthcare workers described these practices in relation to themselves. In other words, in this paper we do not focus on visible practices of dissent that are system-oriented, nor on practices that are aimed at changing other’s behavior, but on less visible practices in which healthcare workers describe a relation to themselves. This analysis was guided by Foucault’s concept of “care of the self”, as a range of practices and techniques to act upon oneself and to steer how one wants to become. Broad themes were initially derived deductively based on this concept. During the coding, subthemes were generated inductively as the coding progressed. Along the coding process both the overarching themes and subthemes were continuously reworked until a satisfactory understanding of the “practices of the self” was reached.

## Results

In this section, we describe different ways in which healthcare workers adapted or changed their own practices when facing supposedly inadequate forms of care in relation to undocumented migrants. It consists of the different techniques that healthcare workers develop and impose on themselves in their search for better ways of caring. Together, these elements provide a detailed description of care of the self in a context of barriers to healthcare.

### Strategic ignorance and controlling affective responses

Many respondents mentioned that, during consultations, they were not aware about the undocumented status of their patients. On further probing, it turned out this was not because the information was not available. Francine,^3^ an experienced doctor at an emergency department of a public hospital, says:

Francine: “But I prefer not to know it at first. It's an advantage for me not to know, I think.”

Interviewer: “Okay. So it's an advantage...”

Francine: “Just... For me, it doesn't matter at the start, it will matter at some point, but at the start... If someone says it [that the patient is undocumented] to me, ok, then I know. But if I don't know, that won't change much. It is true that if I look at the file that I receive from the administrative reception, I can see if indeed they have a mutuality number... I think the advantage of not knowing is that we do not have any a priori. We see the patient without judging too much. So it is important to know that he is in a precarious situation, when it is necessary to make [a treatment], to be more attentive to certain things. But for me, it does not have any disadvantage of not knowing it.”^4^

Francine mentions preferring not knowing about the residence status of the patient at the start of the consultation. She argues that knowing the residence status will affect her a priori judgement. She is aware that information about residence status is collected at the reception, but deliberately avoids knowing this information, and waits for the patient to talk about his residence status, when this interferes with the treatment plan or costs. This practice of actively trying not to know about residence status was mentioned repeatedly among clinicians, both in publics sector and humanitarian settings.

Géraldine, a general practitioner (GP) volunteering in a medical humanitarian non-governmental organization (NGO) in Brussels, in addition to working as a self-employed GP in a rural community, nuances this in the following quote:

“Some (undocumented migrants) do not have access to healthcare, well they do not have the card for urgent medical aid. They do not have access to care whether for reason X, Y or Z, you know. So... I don’t do that anymore, I can't even say if they have papers or not. I don't see them like that. I see that they do not have access to care and that we are trying to put them back into the care system. But I am unable to say if the one who is there, he has his papers or not. Well, you don't see it, it’s just that you have plenty here who have papers but who are... They also dropped out, homeless people, who fell off the public healthcare system.“

Just as Francine, Géraldine is unable to say whether patients have a residence permit (‘papers’) or not. She describes this as something she actively (does not) do anymore. A way of seeing she avoids. However, instead of using the notion of undocumentedness, she uses a different categorization. Together with homeless people, undocumented migrants are seen as people who do not have access to healthcare. Another respondent said: “All these people, are patients I call ‘precarious’.” In other words, deliberately avoiding the categorization of undocumented migrants means using another categorization.

In addition to not wanting to see patients in a certain way, Géraldine also refers to a more sensorial notion of seeing. She mentions the lack of visible, embodied features of undocumented migrants. Luc, a middle-aged GP in a community health center in the public healthcare system, also mentions the role of this visual experience of difference:

Luc: “We treat them [undocumented migrants]. During the wars, doctors, they treat both parties. On battlefields, there are reds and blues, they will treat the reds and blues, that's the ethics of medicine.”

Interviewer: “I see, but on the other hand... in practice... for example, in practice, we see that it is easier to access healthcare as an undocumented child than as an undocumented adult with the same problem.”

Luc: “It's bad. [...] We must protect patients. The human mind notices the differences first. So if I put a white man and a black man next to each other, I notice: ‘Ah, you are white.’ And then black people, we have trouble distinguishing between black people, well, one needs to get used to distinguish their faces, we have all that. And so, you have to rework yourself [Fr: retravailler sur soi], to say: ‘Yes, I see the difference, but is it relevant?’ It is not relevant. And as the difference catches the eye, the first judgement we have, is the difference. So everyone has to say themselves ‘What do I do with this notion that I have about difference?’”

Undocumented migrants are initially described as one of two parties in a conflict. From the quote it is unclear who the other party is, most likely it refers to the Belgian population. Luc refers to a notion of neutrality—a concept from humanitarian and military ethics—to explain why he treats undocumented migrants and the Belgian population alike (as is also advised by the bioethics committee). However, on further probing, a different response is given. Undocumented migration – a state-imposed administrative status – is now described in racializing terms, and with visual metaphors. He describes the experience of difference as something out of his control, it catches his eye; and something universal, we all have that. He describes how to protect the patient, he has actively to reflect on his notion of difference. In order to see the face beyond the difference, he has to rework himself. He explains this as a practice of talking or saying a specific phrase to himself. Reworking himself is described as a process, a way of seeing one has to get used to.

### Rejecting and reworking one’s position in the web of relationships

In several interviews, healthcare workers describe self-awareness of their own position in relation to their patients, and within the wider context of the inaccessibility of the public healthcare system. A second practice of “care of the self” consists in efforts to transform this position.

Marie, a young doctor, working as an employee in the medical humanitarian NGO mentions that the role of her organization is “to provide healthcare to people who are not included, but only for a short period. In fact our role is really secondary.” She mentions that she works “in an organization that actually should not exist as the public system should provide healthcare for everybody.” She explains how she and some of her colleagues adjusted their clinical practice to this:

“So we focus on the most vulnerable people and it is for them, primarily, that we try to achieve access to [public] healthcare. And so we really try, we have to insist on that, that we are there just to try to put them back into healthcare, so we are just covering this time when they are without access to care and... I really see it like that. But this is something that we really have to be firm about with ourselves [Lit: we have to hammer it into ourselves. Fr: on doit nous matraquer] because very quickly, you tend to become a general practitioner of people, their treating doctor. We are there for them during a time when they do not have access to healthcare [...] We made a chart where you see, right now this and that is care in Belgium, and urgent medical aid, and then you have that way, there you have [Name first line services], you have the [Name outreach] and all that. And you have the [Name NGO] and we are just a link to getting there. And so there, we redid this, it's very recent, we redid this map, so the doctor... I always have it in front of me. As a doctor, it’s spontaneous, taking care of them and continuing doing so, you quickly take on a role of general practitioner, you know, you will treat, you take blood tests, he comes back for the result. Well, you quickly put yourself in a... we also tell people. They stay, we tell them: ‘we are helping you, we're just here to help you get urgent medical help and have access to a health centre, have your own doctor, have... being taken care of properly.’”

Marie describes a strong inclination to continue taking care of an undocumented patient in the NGO, once the care relationship has been started. This is true for herself and for her patients. She is aware that this inclination is not in line with her conviction that undocumented migrants should be cared for within the public health care system. Therefore, she tries to bring her clinical practice in line with the intentions of the NGO to include the patients into the public healthcare system as quickly as possible. To make sure this happens, she makes conscious efforts to avoid to become the patient’s treating doctor. She and her colleagues adjust the way they get attached to patients in order to achieve their moral goal of having patient cared for in the public healthcare access. Marie and her colleagues have repeatedly created a sheet to remind themselves of their position in the health system and to retrain the way they practice medicine.

Leen, a social worker in a hospital in Brussels, mentions a different kind of effort to consciously reposition herself when seeing admitted undocumented patients. In the hospital, social workers from the hospital collaborate with social workers from the Public Social Welfare Office (PSWO), who control the distribution of the medical cards. Yet, they have different roles: “The PSWO revolves around the financial. Are they authorized to pay the hospital costs? Are they authorized to give those persons a medical card? While we here, a social worker in the hospital, we don't care about that. Our interests are to ensure that that person is given good aftercare and that he is well cared for within the hospital.” She says about this:

“ I personally have a bit of a problem with that, because I don't want to be associated... Well, I don't want to be directly associated with the PSWO. I am a social worker here at the hospital, and I do what I have to do within the hospital, and she is a social worker at the PSWO. [...] They also have a much more direct way of interviewing the patient. They really insist, while for us it is not necessary to have such a thorough... Their revenues, for example... If people don't tell me how much they earn or whether they work informally, I don’t mind. While they have to go into all the details for their social inquiry. It disturbs me. I try to avoid going to the patient together. I prefer to go alone and they go alone afterwards.”

Leen initially did the interviews together with the social workers from PSWO. However, she started seeing patients alone following several experiences where undocumented patients became afraid, even removing their intravenous line to leave the hospital, after having been interviewed for the social enquiry. She adds that, during that transition, she also started to perform her work with “an attitude that tries to put them at ease.” In order to care for undocumented patients in accordance to her personal norms about care and her institutional role, she had to create a physical distance from colleagues with the same professional background, yet who embodied another, more intrusive way of caring. She also actively aligned her bodily appearance with her caring disposition.

### Cultivating minimalistic medicine, cultivating a different equality

During his ethnographic observations, the author regularly observed healthcare workers experiencing financial and administrative barriers when caring for undocumented migrants. This induced limitations in the available treatment options, the referral options, or access to the most convenient technical investigations. However, to his initial surprise they often did not complain about it. Sometimes healthcare workers even seemed to perk up in such situations. This occurred both in public healthcare services and humanitarian settings, although the limitations were different. During a long interview with Caroline, one of the doctors who the author got to know well during the field work, she revealed:

“I limit myself [Fr. Autolimite], so to speak. By thinking more [...] So the difference, in fact, because it is a philosophy of [name senior colleague], which he is trying to instill, is to say: ‘for this type of patient for whom technical acts will not be paid, will not be billed, and therefore will be paid by the hospital, so by us; it asks us to do the right thing but to think about what is necessary.’ And that is to say... From time to time there’s a tendency in the current teaching of medicine, young doctors, often they make the prescription for the laboratory and they have not yet seen the patient, that's it. Sometimes even a prescription for a scan. And I do the opposite, that is to say to first examine the patient and say to myself: ‘Ok, what is necessary, for this patient? What is really going to change our care? What will guide the diagnosis?’ By trying to practice good medicine without overconsumption, in fact, that's it... in fact, we should do it systematically, there is no reason to do it only for those patients. But unfortunately, right now, and at all levels, it's not just in hospitals... in medicine, they have so much work that it goes faster to prescribe a scan than to to put a hand on the belly of a patient.”

The doctor is limiting the amount of technical investigations she does for undocumented migrants in case of financial implications for both the patient (i.e. high bills and potential debts) and the hospital where she works (i.e. increasing budgetary deficits due to austerity measures and unpaid bills). To the reader, this may initially appear as if she has internalized a notion of substandard healthcare and has surrendered to institutional pressures to minimize costs. However, she describes this as practicing *good medicine*. She implicitly describes the healthcare she provides to undocumented migrants as a traditional approach to medicine and contrasts it with a more recent approach. This current approach is referred to negatively in terms of overconsumption, brief patient contacts, high workload, and high technicality. She expresses nostalgia back to a more hands-on medicine where there is more room for patient contact and clinical examination. For Caroline, healthcare delivery to undocumented migrants creates a situation where she can practice this more traditional kind of medicine. It creates a situation where there is room for diagnostic thinking, priority setting, and efficient use of medical means. She experiences a certain pleasure in the limits she encounters, and in the way she deals with these limitations. It increases the role of her professional judgement and paradoxically she experiences more professional autonomy or freedom to think when being confronted with limitations in healthcare access for undocumented migrants. Put differently, it provides her with an intellectual challenge where she can practice and refresh her professional skills. Later in the same interview, she elaborates further on this:

“This might sound like a very cynical remark, but for the clinician or the professional, they are fascinating patients because they have pathologies that are much more marked. They consult later and therefore, from a professional point of view, it is extremely exciting. But that is very cynical as an answer. Also, obviously we feel much more useful in caring for someone, [...], I think it is much more rewarding but also, yes, rewarding for oneself to treat someone who is in need, who has a big pathology, that nobody else is going to treat in fact, and that we will really help, at least we will try to help, we must remain humble.”

Caroline describes that for her, treating undocumented patients is more exciting, because they present with more severe illnesses (in a context where the available resources are limited) which offers more opportunity to practice one’s skills as a professional. Simultaneously, she expresses awareness that fascination with illness, just as finding professional satisfaction in the ill health of others, is socially not desirable. However, she describes this satisfaction not in relation with others, but in relation to oneself. It transforms the way she feels in her care work. She feels more useful. This experience is not just situated in being competent in the clinical practice, it is also emotionally rewarding; it enables the practice of a virtuous deed, notably helping somebody that nobody else helps.

Géraldine describes a similar active relationship to the self, in order to aspire certain values, when being confronted with limitations in healthcare access, yet she formulates this quite differently:

“We were trained by a doctor who was here before, [name], who stayed four, five years anyway. And somewhere... medicine, he told us to do it the same way, you see really to treat people the same way. And to react as with someone who has access to care, you see, so as not to really make a difference and to ... And that is something that I keep reminding myself. So it is certainly so that there may be some difference, but basically I feel that the quality of the treatments we give them is really equivalent. Well, sometimes we will wait longer for a radiology or for certain things, but basically, you get almost the same thing.”

Similarly to Caroline, she refers to a senior colleague, a role model, who inspired her to practice the way she currently does. Caroline refers to the image of instilling to describe this pedagogic process. Géraldine talks about how she keeps reminding herself of a specific thought to transform the way she acts or reacts. Later in the interview, Géraldine verbalizes this thought as follows: “you have to act *as if* it’s your own patient”. This thought (similar to a mantra) contains a particular paradox; a paradox that is also present in her previous quote. Both quotes simultaneously contain notions of equality and difference. Géraldine expresses aiming for the same outcome, to practice the same way, despite sometimes following a different approach. The use of the infix *as if* suggest awareness of difference and distance and, simultaneously, the mantra urges her to act in an egalitarian way as is deontologically prescribed by the Belgian Advisory Committee on Bioethics [39, 40].

## Discussion

### Techniques of the self

Our findings illustrate a range of practices where healthcare workers establish a relationship with themselves when taking care of undocumented migrants. These healthcare workers identify their own conduct in relation to the accessibility of healthcare services as an object of moral work. The relationship with oneself comprises techniques to guide one’s attention away from the undocumented status, and to master one’s affective responses; it comprises practical mental exercises to remind oneself of one’s role/position in the wider healthcare system and one’s commitment to treat all patients equally; it also comprises practices where one transforms one’s bodily attitude towards undocumented patients; and it compromises transformations in one’s clinical practice towards undocumented migrants. Our respondents describe acquiring these practices as a learning process, inspired by colleagues who function as role models. These practices enable healthcare workers to relate in an ethical way to the existing limitations to healthcare access of undocumented migrants, in ways that sometimes reproduce, sometimes transforms these limitations.

Many of our respondents questioned the use of the concept ‘undocumented migrant’. They express awareness of prejudicial practices. In order to avoid that processes of labelling, categorization or stigmatization influence their clinical judgement towards undocumented migrants, they develop techniques to keep their perceptions and affective responses under control. Such practices of self-mastery have ambiguous effects. First, such a politics of ignorance [42] about residence status avoids recognition of the many barriers in access to healthcare due to undocumented status. As a result, the healthcare worker does not take responsibility to overcome the impact of undocumented status on the availability of treatments; it becomes the patient’s responsibility to addresses this issue himself. Moreover, our findings illustrate that avoiding certain processes of labelling does not protect undocumented migrants from other processes of social categorization. Strategically ignoring patients’ undocumented status goes along with labelling them as so-called precarious patients, but also with a racializing gaze [43] as Luc’s quote illustrates, and also with processes of othering, where migrant patients are portrayed as more fascinating patients or patients with more severe or exotic pathologies as is illustrated by the quote of Francine.

Other practices of the self consist of exercises to remind themselves to reorient their way of relating to undocumented migrants, to direct their attention away from discriminatory government procedures and to suppress their own affective responses. Respondents also reported transforming their body language to embody distance from authorities and approximation towards undocumented migrants. These practices are cognitive exercises, although they clearly also contains aesthetic elements. In this way, respondents showed to be reflexive about the strategic importance of care practices. These practices of the self function as a way of avoiding that care practices support the scrutiny of undocumented migrants, or the exclusion of undocumented from the public healthcare system. Moreover, earlier research shows that these hardly visible forms of resistance went beyond mere practices of the self, and also consisted in challenging healthcare workers that formed an immediate barrier to healthcare access, as expressed through Foucault’s notion of counterconduct [30, 37].

However, some healthcare workers also express a sense of meaning in the limitation of the available healthcare services, or in limiting them for themselves. They focus on training themselves by developing, or rediscovering, clinical skills that allow them to take care of undocumented migrants within these limitations. They express an aesthetic experience of excellence or feeling useful, together with feelings of excitement and fascination. Interpreted this way, these care practices (as practices of the self) rather take the form of finding pleasure, freedom or feelings of virtuousness in one’s capability of self-limitation, or one’s ability to comply with a norm imposing limitations [13]. By undertaking conscious effort to practice a different, more minimalistic kind of medicine, these healthcare workers cultivate an ideal of *good medicine* that is associated with parsimony, moderation and professional excellence. In other words, these self-practices transform their care work into a profession that carries certain aesthetic qualities without subverting the structural constraints imposed on them.

### Questioning the biopolitical subject

Our findings show that practices of the self not necessarily always should be interpreted as resources for resistance, or counter-conduct, in response to restrictions in healthcare access. These findings resonate with Mahmood’s [22] interpretation of Foucault’s work, questioning the tendency in poststructuralist scholarship to read practices of the self only in terms of resisting the dominating powers. The stress some respondents put on the pleasure they found in coping with the experienced limitations in healthcare access shows that these practices can also be focused on the individual satisfaction; putting particular emphasis on self-cultivation [45]. Nevertheless, we argue that our findings challenge the current views on care of the self in biopolitical analyses of humanitarianism. Scholars in the field of humanitarianism have understood “care of the self” mainly as a practice of healthcare workers procuring their own needs [37] and a way in which healthcare workers can fashion themselves as more enlightened persons [38]. In this view (humanitarian) care work produces subjectivities that sustain, conform and align with practices of government [38]. Contrarily, our study illustrates how healthcare workers can (trans)form themselves as an active ethical subject in a context of humanitarian government of migrants. In the context of our study self-cultivation also generates a new subjectivity, notably one of healthcare workers aspiring to practice (as) excellent medicine with less means. In line with Foucault’s views, the subject can be free within the constraints of the government, and create new forms of subjectivity [13, 17, 46]. This contrasts with the passive and docile subject set forth in biopolitical analyses of humanitarianism.

As useful as biopolitical analyses are for understanding how power works through humanitarianism and care, the concept (and the way it is applied) also has important analytical constraints. A biopolitical analysis obscures how those working in the health care sector actively relate to efforts to control migrants. It obscures how healthcare professionals function as subjects in the context of these power relations, and how they can acquire a certain independence towards processes of normalization and surveillance. Within the wider society, those providing care do not always hold a powerful position [47].The majority of the jobs in migrant care services, are filled by women and “‘feminized’ others”, with relatively low wages and substantial voluntary work [48, 49]. These jobs are often valued in feminized qualities such as empathy, serving,… Their activities are often devalued or marginalized [49]. Biopolitical analyses of healthcare to migrants somehow reproduce this marginalization by framing healthcare workers as merely being obedient instruments of humanitarian government, merely interested in “caring for their disconcerted selves” [38].

Moreover, such analysis also has normative constraints. In a biopolitical analysis of humanitarianism nobody seems to be doing what they say; or what they do never has the intended effect. As a result, moral sentiments associated with care for a “distant other” are often regarded with reluctance, mistrust, or sheer cynicism [50]. This can nourish apathy, distance and cynicism among healthcare workers. A range of uncertainty and/or ugly feelings become associated with the (humanitarian or deontological) imperative to care in ways that benefit distant others [50, 51]. Biopolitical analyses of humanitarianism take a largely negative and critical orientation towards care practices harboring moral aspirations, without taking responsibility for what should replace them.

### The (ir)relevance of counterconduct

Many respondents described their practices as a way of challenging institutional practices. Some tried to eliminate individual subjective prejudices by avoiding the use of state-imposed categorizations. Others challenged the restricted healthcare for undocumented migrants by adopting specific practices of the self in interactions with this particular group. In other words, the practices present a mixture of universalizing and categorical approaches [36]. In both cases, these practices of the self can be understood in terms of activist practices of healthcare workers who are self-aware of the power relations in which they are involved. However, several critics of Foucault question whether practices of the self are well fit as instruments for challenging the biopolitical effects of care, even when intended as such [52]. They argue that practices of the self do not lead to engagement in collaborative political projects with others, nor in strategies to counter the depoliticizing effects of biopower [45, 53]. Respondents did indeed describe these practices of the self as very personal. Similarly to what our respondents described about more visible practices of dissent, they describe practices of the self as an individual ethical choice. They generally expressed tolerance, or even indifference, towards colleagues who make other choices. In this regard, practices of the self bear an individualist connotation. The responsibility for ethical clinical practice towards undocumented migrants is transferred to the individual healthcare worker.

In addition to this, practices of the self were mainly described by respondents in clinical settings. They were described by healthcare workers, working both in humanitarian organizations and the public healthcare sector. They were mentioned far less by receptionists and social workers in social welfare services, where the primary focus is on administrative procedures, and where clinical considerations are less present. Healthcare workers trying to behave ethically in clinical contexts do not just rely on the application of abstract principles, codified ethical standards or laws [31]. Foucault argued that practices of the self are mainly an important element in systems of codes that are rather rudimentary. He described it as an ethics that was somehow disconnected from strict prescriptive systems, defining stringently what is permitted and forbidden [13]. Professional codes, such as modern medical ethics for example, do not attempt to provide ready-made answers in the context of clinical practice [54]. This provides more room to individual healthcare workers to develop ethical practices in relationship to oneself.

However, earlier research showed that undocumented patients negotiating healthcare access mainly interact with receptionists who operate in a much more prescriptive and regulated work environment [44, 55]. In other words, these practices of the self described by clinicians might take place in a clinical setting that is already foreclosed. Foucault also pointed to these complexities of an ethics relying on practices of the self: he mentioned that practices of the self can become integrated into wider structures of domination, resulting in a versatile equilibrium between practices that assure domination and practices through which the self modifies itself to become a different kind of subject [56].

### Care of the self … or care?

Our findings show that practices of care of the self also impact on the care relationship. One respondent, for example, mentioned attaching in a different, more tempered way to ensure that undocumented patients can access the public system. Critics of Foucault have argued that the individualist character in Foucault’s account of the care of the self is not only in contrast with political collaborative projects, but with collective practices in general [45]. Meyers argues that care of the self detaches a person from their relations with others and furthers the segmentation of individuals. The care relationship is a social relation that involves interaction, dialogue, mutuality, and reciprocity. Allen [57] finds that the relations with others in Foucault’s account of care of the self are unsatisfactory as a basis for ethical practices as they do not establish responsiveness, mutuality and reciprocity. In line with feminist views, she states that ethical practice should take particular relations with concrete others as a starting point.

Foucault [12] described practices of the self as a communal activity, proposed by one’s society or social group and requiring the support of friends and family. He also stressed the role of lessons of a mentor in acquiring practices of the self. Similarly, Mayes [58] showed that practices of the self, resisting against the body norms in all kind of lifestyle-advices, are a form of collective resistance, involving relations with others and guidance by a ‘master’ figure. Papadimos et al. [59] argue that practices of the self in mentoring and medical teaching also implies care of the political life, requiring engagements in hospital committees and support of colleagues. In the interviews, several of our respondents refer to the importance of senior colleagues as role models in developing these practices of the self. The quote form Marie also illustrated that such practices can be worked out together with colleagues and can be part of a wider professional culture. Although this implies a relation with others, these others largely seem to involve members of the same social group, in our case notably fellow healthcare workers.

Moreover, practices of the self, aimed at providing equal quality of healthcare for undocumented migrants, are not easily reconcilable with realities of competing demands and requirements of priority-setting in healthcare settings. Day-to-day care practices are characterized by many relationships with individuals with varying degrees and kinds of dependence [60]. Furthermore, biomedical science (and socialization of junior healthcare workers within medical institutions) relies heavily on categorical thinking, allowing healthcare workers to differentiate between subgroups of patients in order to standardize the management on the level of subgroups and provide care in an efficient way [14]. The ethical conduct described by our respondents is focused on avoiding or modifying the use of one specific category. This conduct is disconnected from the wider context that is characterized by processes of categorization and inherent limitations in the possibilities for responsiveness. Therefore, the ethical relevance of these practices of the self remains ambivalent.

## Conclusion

Ethics defined as care of the self broadens the repertoire of frameworks available for understanding care practices of healthcare workers to undocumented migrants. It offers a framework for understanding ethics as a practice that is independent of the approach of traditional professional ethics and formal codes of ethics. Moreover, it offers a framework for moving beyond biopolitical analyses understanding healthcare workers as instruments in processes of categorization, surveillance of migrants and humanitarian government. Instead, this framework puts the subject of the healthcare worker at the forefront as an active ethical subject, experiencing freedom in the constraints of being a subject. It locates the ethical in practical mental exercises of healthcare workers to be self-reflexive, to master themselves and to rework one’s clinical practice. This involves healthcare workers acting upon themselves, thus creating an active relation to the self.

Our analysis of these practices of the self highlights the ambivalence of the role that practices of the self play in developing ethical clinical practice towards undocumented migrants. First, practices of the self can be interpreted as subtle forms of resistance to prevalent forms of categorization and government of migrants. However, the political work remains at the individual level and does not lead to engagement in the justice struggles of migrants to change structural limitations in healthcare access. Secondly, practices of the self also have an aesthetic dimension which are expressed in terms of excellence, but also selflessness. In the settings we described, achieving these aesthetic values is closely linked to realizing ethical values like equality. However, such aesthetic understanding of ethical practice can also be considered as a self-congratulating activity of self-fashioning in which others, and more precisely patients and care relationships, are of secondary importance. Therefore, practices of care of the self of healthcare workers cannot be considered as a distinct undertaking. They should not be disconnected from more collective efforts to defend health rights and to provide equal access to healthcare; nor should they be disconnected from attention to, relationships with and care about concrete others.

## Supplementary Information

**Additional file 1:** Interview guide healthcare workers.

## Data Availability

The data for this project are confidential. Therefore, there are no research data in a public repository for this submission. The informed consent form of the in-depth interviews explicitly excluded public sharing of the data. The data were anonymized in such a way that confidentiality was guaranteed. Data processing was done in accordance with the relevant Data Protection and Privacy legislation.

## Abbreviations

B.U.N.: Belgisch Uniek Nummer [English: Unique Number of registration in Belgian Medical Ethics Committee]
GP: General Practitioner
NGO: Non-governmental Organization
PSWO: Public Social Welfare Office
UMA: Urgent Medical Aid
UZ: Universitair Ziekenhuis [English: University Hospital

## Footnotes

1 When writing about the later work of Foucault, I refer to the moment of publication. Chronologically Foucault developed the notion of counter-conduct earlier than History of Sexuality Vol 2 & 3, yet the lectures where he outlined this concept were only published much later. Therefore, this concept is considered to be part of his later work.

2 Foucault’s concept “care of the self” is not synonymous to the concept of self-care, that is also often used in healthcare contexts. There is a variety of definitions of self-care [24]. Self-care definitions usually involve a spectrum of care activities ranging from practices for remaining in good health on one’s own to practices for complying with professionally prescribed treatments in collaboration with health professionals [24, 25].

3 All names are pseudonyms for reasons of anonymity.

4 Translated from French. All quotes have been translated from French or Dutch by the author.
