# Supplementary material for: Beyond biopolitics: the importance of the later work of Foucault to understand care practices of healthcare workers caring for undocumented migrants
Source: BMC Med Ethics. 2021 Nov 27;22:157. doi: 10.1186/s12910-021-00726-z (PMC8627089; doi:10.1186/s12910-021-00726-z)
Supplement: Supplementary file 1 — Additional file 1: Interview guide healthcare workers. [file 12910_2021_726_MOESM1_ESM.docx]

Questionnaire & detailed interview guide healthcare workers

Location: ...............................

Date: .....................................

**Before the interview : Demographic data.**

Gender: m/v/x

Age: .............

Employment status: .............

(Highest) level of education: .............

Nationality: .............

How many times per week do you consult undocumented migrants (cf. infra)? .............

**Introduction questions**

How would you describe your role in the health care system? How did you get into this role/how did you start working here?

**Transition questions**

How would you explain the procedure ‘Urgent Medical Aid’?

How does the procedure ‘Urgent Medical Aid’ apply to undocumented migrants?

When is a patient undocumented for you?

How often do you come into contact with undocumented migrants as a healthcare worker? Why do you think you have many/ few people without legal residence as patients?

**Core questions**

Do you notice differences in the health and differences in healthcare access between residents and individuals without legal residence status?

Can you give some examples where undocumented status impacts on your clinical decision making?

Possible probing questions:

What situations have you remembered? Did you ever have the feeling after a consultation with a patient without legal residence: 'I should have done this differently...'?

Points of attention during healthcare provision to patients without legal residence status? Why do you pay attention to this?

Problems or conflicts during healthcare provision to patients without legal residence status? What is the cause of these problems or conflicts? What are the consequences or health effects?

Hypothetical questions (in case the respondent reports no previous experiences):

1. Patients have to contact various care institutions and actors in order to obtain access to UMA, they also have to keep visiting them repeatedly to keep their medical card. How do you deal with this complexity in case of urgent medical problems?
2. What do you do when undocumented patients ask you to make a prescription/attestation in the name of a family member who is legally residing in Belgium?
3. What do you do when you see a patient with a medical problem that you can and want to treat, but whose administrative file (UMA) is not yet processed, is not capable to pay him/herself for the treatment you propose? What happens if treating that patient has financial implications for the institution you work for?

As a healthcare worker, how do you deal with situations where the healthcare legislation for undocumented migrants is not in line with your deontology or your personal convictions?

How do you deal with other elements in the situation of undocumented migrants that you cannot change (e.g. poverty, unstable living conditions, risk of deportation, linguistic barriers)?

**Concluding questions**

Do you want to add something? Any further remarks?
